# Parental Behavioral Control and Bullying and Victimization of Rural Adolescents in China: The Roles of Deviant Peer Affiliation and Gender

**DOI:** 10.3390/ijerph18094816

**Published:** 2021-04-30

**Authors:** Xu Chen, Ling Li, Gangwu Lv, Hui Li

**Affiliations:** 1 Center for Education Policy, Faculty of Education, Southwest University, Chongqing 400715, China; chun1122@email.swu.edu.cn; 2 Resources and Environment College, Southwest University, Chongqing 400715, China; 3 School of Education, Macquarie University, Sydney, NSW 2109, Australia; philip.li@mq.edu.au

**Keywords:** parental behavioral control, deviant peer affiliation, bullying, victimization, rural middle school, gender differences

## Abstract

Bullying and victimization (BAV) have been widely studied, but the potential mechanism of parental behavioral control (PBC) on bullying and victimization in Chinese adolescents has not been explored. This study aimed to examine a moderated mediation model for the association between PBC and BAV mediated by deviant peer affiliation (DPA) and moderated by gender. A total of 3779 adolescents (*N_boy_* = 1679, *M_age_* = 14.98 years, *SD* = 0.95) from southwest China has completed the Peer Bullying, Peer Victimization, PBC, and DPA questionnaires. The results indicated that: (1) PBC significantly predicted adolescents’ BAV (−12%); (2) DPA mediated the effect of PBC on BAV only for those adolescents who were both bullies and victims; (3) the mediating role of DPA was moderated by gender only in the relationship between PBC and victimization, with a relatively stronger effect in girls than in boys.

## 1. Introduction

School bullying is a repeated act of harm, including physical bullying, social manipulation, verbal bullying, attacks on property, and cyberbullying [1,2,3,4]. Many students have been suffering from school bullying, and some victims have even become bullies [5,6,7]. However, being victims, bullies, or victims-and-bullies would damage their subsequent mental health and social adjustment and even lead to an increased risk of later criminality [1,5,6,7]. To break this vicious circle, parents and peers should engage in the fight against school bullying, as many studies have reported the influential roles of parents or peers in bullying and victimization during adolescence [8,9,10,11,12]. However, few studies have thoroughly explored the potential mechanisms underlying the path from parental behavioral control to school bullying and victimization [13,14]. In particular, Chinese parents are well known as ‘tiger moms’ and ‘panda dads’ [15]; thus, they tend to have more parental behavioral control, which will inevitably impact school bullying and victimization in Chinese adolescents. This unique impact has not been empirically explored. To fill this gap, this study explored how Chinese parents’ behavioral control of making friends impact their children’s bullying and victimization and what are the roles of deviant peer affiliation and gender in this relationship. The findings will provide educational implications for school bullying prevention and evidence-based suggestions for parental guidance.

### 1.1. The Association between Parental Behavioral Control and Bullying and Victimization

School bullying means the repeated harmful behavior of one or more students against peers, such as assault, abuse, spreading rumors, etc. [1,2]. It includes five types in the existing literature: physical bullying, social manipulation, verbal bullying, attacks on property, and cyberbullying [3]. A victim of bullying is a student who was attacked and suffered physical or psychological harm when bullying occurred at school [16,17,18]. Students who have conducted bullying and have been bullied by their peers are considered bullies/victims [6]. The interaction between parents and their children is closely related to bullying and victimization. For example, recent studies on Asia students have found that parental knowledge, punitive parenting, parental attachment, parental warmth, and acceptance could predict adolescents’ bullying and victimization [8,10,12,19].

Parental behavioral control is an integral part of parental control, which refers to parents’ supervision and interference in their children’s psychological or behavioral activities [20,21]. Psychological control includes getting involved, triggering guilt, and withdrawing love, while behavioral control includes the rules, regulations, and restrictions set by parents for their children [21,22,23]. Some researchers reported that psychological control hurt children’s function, whereas behavioral control positively affected [20,23]. In particular, parental psychological control could reinforce adolescents’ negative behaviors, such as aggression, drug use, and bullying [20,23,24]. However, behavioral control could reduce such behaviors to a certain extent [23,25]. Moreover, Li et al. found that parental behavioral control could effectively reduce Chinese adolescents’ peer victimization through self-control, but parental psychological control would make them more bullying [26]. Based on these, we inferred that parents’ behavioral control over adolescents’ making friends negatively correlated with bullying and victimization and might also predict bullying and victimization by other factors. Thus, we proposed Hypothesis 1: that parental behavioral control could predict bullying and victimization of adolescents.

### 1.2. The Association between Deviant Peers Affiliation and Bullying and Victimization

Deviant peer affiliation refers to adolescents’ selective affiliation with their peers with serious problem behaviors, including cheating, aggression, and substance abuse [27,28]. There are many causes of deviant peer affiliation in adolescents [29,30,31]. One of them might be the poor parent-child relationship. A recent study has found that the low quality of the father-child relationship could cause more frequent deviant companions in Chinese adolescents [31]. As the group socialization theory believes, the peer group is an important environment for children’s socialization development [32]. They have to follow the same rules and behavioral patterns to get accepted by the deviant peers [27,32,33]. In return, deviant peer affiliation would undoubtedly cause serious externalization behavior problems, such as drug abuse, aggressive behavior, and bullying [29,34,35,36]. In other words, parent-child interaction, such as parental behavioral control, is likely to arouse school bullying via deviant peer affiliation.

However, exposing oneself to deviant peers can also easily make adolescents a victim. Deviant peer affiliation has been found highly correlated with peer victimization and has been identified as one of the causes of bully victimization in Korean adolescents [19]. However, this finding did not effectively distinguish the different identities of bullies such as bullies, victims, bullies-victims, and non-status. In addition, this finding might not necessarily apply to Chinese adolescents in rural schools. Therefore, it is urgently needed to explore the predictive power of deviant peer affiliation in identifying Chinese rural students’ groups and status. Based on the above research findings, we hypothesize that deviant peer affiliation might be a mediator between parental behavioral control and bullying and victimization. Accordingly, we proposed Hypothesis 2 for this study: deviant peer affiliation might mediate the relationship between parental behavioral control and bullying, victimization.

### 1.3. Gender Difference in Deviant Peers Affiliation, Bullying, and Victimization

Gender differences in deviant peer affiliation have been widely investigated. Many studies confirmed that boys were more likely to have deviant peer affiliation than girls [37,38]. A similar finding has also been reported in Chinese samples [39,40]. The arguable point is whether girls or boys are likely to be affected by deviant peer behavior, as the existing findings are not consistent [41]. This inconsistency might be caused by the differences in samples, contexts, and cultures, thus warrants more studies in different contexts such as rural schools in this study.

Many studies have also found gender differences in school bullying [39,42,43,44,45]. Generally speaking, boys are more likely to bully and become bullies than girls [44,45]. In terms of types of bullying, boys are more likely to bully physically than girls. In contrast, girls tend to use more indirect bullying, such as speaking ill of others behind their backs, destroying peers’ relationships to isolate them from the peer group, and destroying peers’ personal belongings [42,45]. Some recent studies have found that gender might be a significant moderator in the relationship between deviant peer affiliation and adolescents’ problem behavior and risk-taking behavior in Chinese adolescents [39,43]. Bullying is closely related to external problem behaviors, and both are negative aspects of adolescent behavior development [46,47,48]. Based on this, we inferred that gender might also be a non-negligible moderator between deviant peer affiliation and adolescents’ bullying.

Gender difference in bullying victimization has also been widely reported. A meta-analysis study has concluded that boys in adolescence were more likely to become bullying victims than girls [49]. Research on Korean adolescents also found that girls’ bullying victimization was highly correlated with deviant peer interaction than boys’ [50]. Although it could be inferred that gender may be a moderator between deviant peer affiliation and bullying victimization, there was still insufficient evidence to support the view on how it works. Therefore, we proposed Hypothesis 3 for this study: gender might moderate the mediating role of deviant peer affiliation in parental behavioral control and bullying and victimization.

### 1.4. The Context of This Study

China is a collectivist society with a strong Confucianism heritage that highly values parental influences and peer relationships [51,52,53]. Chinese parents generally supervise their children more strictly because of their high expectations [51]. In this context, deviant peer affiliation or parental behavioral control might have a relatively strong impact on school bullying and adolescents’ victimization. Thus, it deserved empirical studies. In addition, rural adolescents in China are more likely to experience school bullying, and more than half of rural students had ever been bullied by peers [54,55]. According to the National Statistical Report, 94.96 million students lived in rural China in 2016. In other words, there might be more than 47 million rural students suffering from school bullying. Therefore, it is urgently needed to reveal the prediction mechanism of parental behavioral control on school bullying and victimization in rural China, which is of great significance in theoretical development and practical improvement of anti-bullying in school.

Recently, a study on rural Chinese adolescents [52] found that boys reported significantly higher bullying victimization than girls. School belonging and academic engagement might be the serial mediators between bullying victimization and academic achievement in boys and the whole sample models. However, the mediation model for girls differed from that for male ones, as academic engagement did not predict academic achievement in girls. These findings indicated that gender might play an important moderating role in the sample of Chinese rural adolescents. However, this study did not clearly distinguish between bullies, victims, and bullies-victims. The current study was dedicated to understanding the potential paths between parental behavioral control and bullying, victimization in Chinese rural adolescents. Based on the literature review above, we hypothesized that:

**Hypothesis** **1** **(H1).** *Parental behavioral control could directly predict school bullying and victimization;*

**Hypothesis** **2** **(H2).** *Deviant peer affiliation might play a mediating role between parental behavioral control and bullying/victimization;*

**Hypothesis** **3** **(H3).** *The mediating role of deviant peer affiliation might be moderated by gender.*

## 2. Method

### 2.1. Participants

This study was part of a large research project commissioned by Q city’s education department, located in southwestern China. The rural area is economically disadvantaged and has 106 junior secondary schools altogether. Only 102 schools accepted our invitation and consented to participate in this project. One Grade 9 class was randomly sampled from the 102 schools, and all the students of these 102 participating classes were invited to join this survey study. Finally, about 3779 students consented and completed the survey, including 1679 boys (44.9%) and 2066 girls (54.7%). The average age was 14.98 years old (*SD* = 0.95).

### 2.2. Measures

*Bullying and Victimization*. Multidimensional Peer Bullying Scale (MPVS-RB) and Multidimensional Peer Victimization Scale-Revised (MPVS-R) were developed by Betts, Houston, and Steer (2015). These two scales had been used to measure Chinese adolescents’ peer bullying and peer victimization in previous studies [52,55]. Both of the two scales contained 20 items and five dimensions (physical, social, verbal, property, and electronic bullying or victimization). Participants were asked to answer each items from 0 to 4 (0 = never, 1 = once, 2 = twice, 3 = three times, and 4 = more than 3 times). In this study, the Cronbach alpha coefficients were both 0.95 for MPVS-RB and MPVS-R.

*Parental Behavioral Control*. Jaccard et al. used the adolescent perceptions of parental control to measure parents’ control about adolescents’ behaviors [56]. Items such as “whether parents let you make your own decisions about the people you hang around with?”, “how much television you watch” and so on. Participants need to answer yes or no on each item. Wang et al. developed a Chinese version based on that of Stattin and Kerr [57,58]. This study aimed to explore parents’ behavior control about their children making friends, so we finally adopted one item from the Chinese version to let adolescents evaluate the degree of their parents’ control when choosing friends. The item reads as: “parents will clearly tell me who could be my friends and who cannot”. Responses ranged from 1 to 4 (1 = never; 2 = sometimes; 3 = often; 4 = always). This one-item measurement has been widely used in previous studies and proved reliable and valid [59,60,61].

*Deviant Peer Affiliation*. Self-assessment had been used by Elliott et al. to assess 15 kinds of delinquent behaviors [61]. This was then adopted to assess Chinese adolescents’ deviant peer affiliation and proved reliable [23,39,62]. Consisted with the current research’s purpose, 7 deviant behaviors related to adolescent bullying were selected from the 15 behaviors mentioned in the study of Elliott et al. These 7 behaviors included playing truant, violating school discipline, fighting, smoking or drinking, going to an internet cafe, having a girlfriend or boyfriend and dropping out of school. Responses ranged from 1 to 3 (1 = nobody; 2 = one or two; 3 = three or more). In this study, the Cronbach alpha coefficient was 0.90.

*Gender, Age, and Number of Friends*. Multiple choice questions and open answers were used to collect demographic information. The items are: “what’s your gender” (biological gender, 1 = male, 2 = female), “when is your year and month of birth”, and “how many close friends you have” (1 = none; 2 = 1 or 2; 3 = 2 to 5; 4 = 5 to 10; 5 = more than 10).

### 2.3. Procedures

The ethical procedures of human research are followed in the process. First, this study was carried out with the approval of the first author’s university and the research site’s educational authorities. Second, the principals of all the participating schools were invited and have consented to join this study. Third, all the Grade 9 students of the participating schools were invited to participate in this study; and they were allowed to quit it without any penalties. Fourth, the trained researchers came to the class and briefed them about this study and their rights. All the participating students completed this survey with informed consent.

### 2.4. Hypothesized Model and Data Analysis

**Figure 1:**
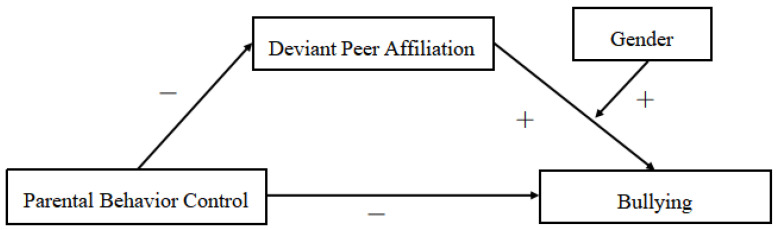
The proposed moderated mediation model for bullying.

**Figure 2:**
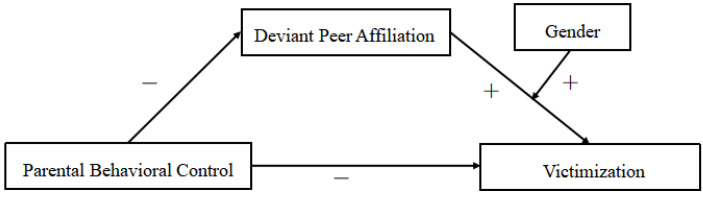
The proposed moderated mediation model for victimization.

Based on the hypotheses proposed, we built a moderated mediating model with bullying and victimization as the dependent variables, respectively (see Figure 1 and Figure 2). Accordingly, the following questions guided this study:

1. Does parental behavioral control directly impact school bullying and bullying victims in rural adolescents?
2. Does deviant peer affiliation mediate the relationships between parental behavioral control and bullying and victimization?
3. Does gender moderate the influence of deviant peer affiliation on bullying and victimization, and how does it moderate this influence?

To address the above questions and to test the hypothesized models, we have conducted data analysis on SPSS25.0 (International Business Machines Corporation, Armonk, NY, USA) using PROCESS 3.2. Firstly, descriptive analyses, including the frequency, mean value, standard deviation, and correlation coefficient, were conducted to clear the basic information and the relationship among this study’s main variables. Secondly, to determine whether there were differences among the four groups in parental behavioral control, the number of close friends, and deviant peer affiliation, we performed a one-way ANOVA to test these latent differences. Finally, logistic regression and PROCESS analyses were conducted to identify the mediating role of deviant peer affiliation and the moderating role of gender to test the proposed models in this study.

## 3. Results

### 3.1. Descriptive Statistics

As shown in Table 1, most participants reported bullying or being bullied by their peers at least once in one way, such as punched or being punched. Further analysis found that some adolescents had only bullied others and no been bullied (bullies group, 10.60%), some had only been bullied (victims group, 12.80%), many had both bullied and been bullied (bullies-victims group, 65.40%), and some had neither bullied nor being bullied (no-status group, 11.20%). Accordingly, they were classified into four groups: bullies group (*n* = 401), victims group (*n* = 484), both bullies-victims group (*n* = 2471), and no-status group (*n* = 423). For details, see Table 1.

Table 2 presents the means, standard deviations of parental behavioral control, deviant peer affiliation, bullying and victimization, and the correlations among this study’s main variables. The correlation results indicated that parental behavioral control was negatively correlated with deviant peer affiliation, bullying, and victimization (*p* < 0.05), as was gender with these three variables (*p* < 0.05). Deviant peer affiliation has a remarkable positive correlation with bullying and victimization.

One-way ANOVA was conducted to explore the between-group differences in parental behavioral control, number of friends, and deviant peer affiliation. As shown in Table 3, parental behavioral control of the bullies-victims group was significantly less than that in the other three groups (*F*_(3, 3775)_ = 5.63, *p* < 0.01). Still, the affiliation of deviant peers was significantly higher than that in other groups (*F*_(3, 3775)_ = 7.98, *p* < 0.001). Therefore, it could be seen that adolescents who had both bullied peers and been bullied by their peers were less constrained by their parents about how to choose their friends. Although they made some close friends, most of these friends might have many deviant behaviors. Compared with the bullies, bullies-victims, and no-status groups, the number of friends in the victim group was the least (*F*_(3, 3775)_ = 63.98, *p* < 0.001). Those adolescents who had only suffered peer bullying were partly related to the lack of friends, which made them lonely and more likely to be the targets of peer bullying (Table 3).

### 3.2. Predicting the Grouping of School Bullying and Bullying Victimization

Multivariate logistic regression was conducted to test the predictive power of parental behavioral control, deviant peer affiliation, and gender on their children’s grouping of school bullying and victimization. In this analysis, the non-status group was set as the reference category. Gender was encoded as 1 and 2 and then brought into the regression (1 = boy, 2 = girl). As shown in Table 4, the regression results indicated that the three factors had no significant predictive power (*p* > 0.05). In contrast, parental behavioral control and gender could negatively predict students’ belonging to the bullies/ victims group (β = −0.13, *p* < 0.05; β = −0.45, *p* < 0.001), and deviant peer affiliation positively predict this grouping (β = 0.60, *p* < 0.001). These results indicated that: (1) the more parental behavioral control, the fewer risks to be bullies-victims (−12%); (2) girls had significantly fewer risks to become bullies-victims (−36%); and (3) deviant peer affiliation would significantly increase the risk of being bullies-victims by 83%.

### 3.3. Deviant Peer Affiliation as a Moderated Mediator

Model 14 in PROCESS was used to examine the hypothetical mediation models in bullies-victims groups. The results indicated that parental behavioral control’s direct effects on bullying and victimization were not remarkable (β = −0.01, *p* > 0.05, 95% CI contained 0). Still, the indirect effect of deviant peer affiliation was significant in predicting bullying and victimization for the bullies/activities group (β = −0.02, *p* < 0.05, 95% CI did not contain 0). These results indicated that deviant peer affiliation played a complete mediating role in the relationship between parental behavioral control and bullying, victimization. Although parental behavioral control did not directly reduce their bullying behavior or victimization, it might prevent them from bullying or being bullied by reducing their communication with deviant peers for adolescents being both bullies and victims. In addition, the interaction between deviant peer affiliation and gender positively predicted the victimization (β = 0.09, *p* < 0.05, 95% CI did not contain 0), indicating that the mediating role of a peer was moderated by gender (see Table 5 for details).

**Figure 3:**
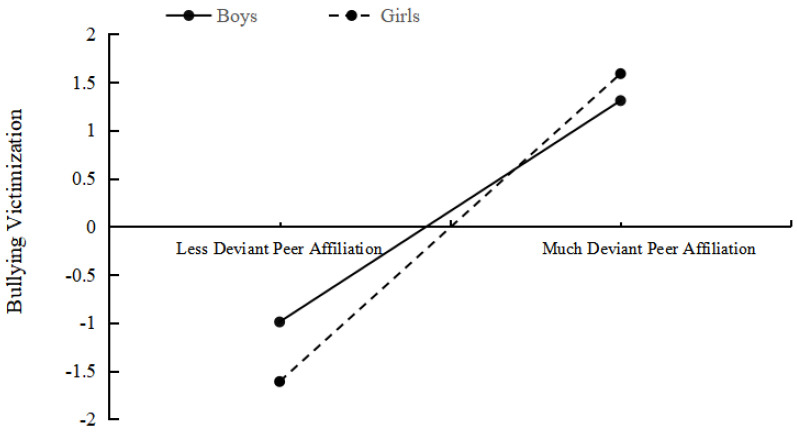
Interaction effect between deviant peer affiliation and gender on victimization.

**Figure 4:**
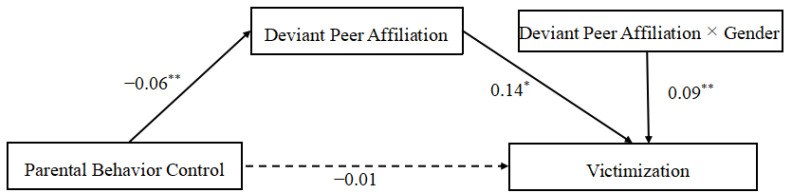
Deviant peer affiliation as a moderated mediator between parental behavioral control and victimization. Note. * *p* < 0.05, ** *p* < 0.01.

In order to clearly understand how gender plays a regulatory role, we conducted the simple slope test to compare the difference between boys and girls. The result of the *t*-test showed that the simple slope of victimization by deviant peer affiliation was significant both in boys and girls (*t* = 0.74, *p* < 0.05; *t* = 10.04, *p* < 0.05). The more deviant peer affiliation boys reported, the more victimization they got, as do girls. However, the size of the two slopes was different between boys and girls. As shown in Figure 3, victimization reported by boys and girls both increased as deviant peer affiliation increased. Still, the speed of this increase in girls was faster than that in boys. These results indicated that deviant peer affiliation played a moderated role in the relationship of parental behavioral control and victimization for the adolescents who bullied peers and were bullied by peers (see Figure 4 for details).

## 4. Discussion

As the first exploration of the predictive power of parental behavioral control and deviant peer affiliation in bullying and victimization among rural students, this study found a significant group and gender difference. The more parental behavioral control, the fewer risks to be bullies-victims, whereas deviant peer affiliation may significantly increase the risk of being bullies-victims. The predictive effect of deviant peer affiliation was also moderated by gender. This section will discuss these findings, their limitations, and the implications.

### 4.1. Prediction of Parental Behavioral Control on Bullying and Victimization

This study found that parental behavioral control could not directly predict school bullying and bullying victimization in all the groups except for bullies-victims. Compared with the no-status group, the more parental behavioral control, the fewer risks to be bullies-victims (−12%). This finding suggested that adolescents’ friends’ parental behavioral control could reduce the risks of being bullies-victims in rural adolescents. In this study, parental behavioral control included regulating adolescents’ making friends; thus, it directly affects the deviant peer affiliation. This implies that by preventing adolescents from communicating with deviant peers, Chinese parents could indirectly reduce the risks of becoming bullies-victims. Bullies-victims have double identities and are at high risk of being referred for mental or behavioral problems than other groups, such as being more anxious, temperamental, and socially isolated [63,64,65]. This is consistent with the findings of studies in other countries, which found that the mental health of adolescents at risk was relatively poor [66,67]. This finding has important implications for preventing school bullying in rural areas: parental behavioral control should be encouraged and guided to effectively reduce students’ affiliation with deviant peers and then to prevent the occurrence of bullying and being bullied.

### 4.2. Deviant Peer Affiliation as a Mediator

This study found that deviant peer affiliation would significantly increase the risk of being bullies-victims by 83%. The rapid development of self-consciousness in adolescence, in conjunction with the boarding status in rural schools, has made the rural students more sensitive to their peer influence [56,65]. According to the group socialization theory [32], peers and parents might play a different role in school bullying and victimization. Peer influences are direct and straightforward, whereas parental influences are indirect and far-reaching, thus mediated by deviant peer affiliation [56]. Thus, it is understandable that deviant peer affiliation was found as a complete mediator between parental behavioral control and peer victimization. This finding implies that replacing deviant peer affiliation with healthy peer relationships might reduce bullies and victims in Chinese adolescents.

### 4.3. Gender as a Moderator

This study confirmed two different models for male and female adolescents in rural China. In particular, girls who made deviant peers suffered less than boys. Still, with the increase in deviant peer interaction, the risk of girls’ bullying gradually increased and exceeded that of boys. This finding is consistent with that girls were more likely to be influenced by deviant peers than boys by Emler et al. (1987). The gendered path found in this study is partially supported by a recent study in China [52], which found that gender played a significant moderating role in predicting bullying victimization in rural adolescents. The role of deviant peer affiliation and gender in parental control and victimization in China has again proved the importance of the family and school context to adolescents’ mental health, which echoes the findings of Escobar et al. [68].

### 4.4. Limitations and Implications

It is undeniable that there were some limitations in this study. First of all, this study was a survey study that simply invited the students to complete the questionnaires. Future studies should invite their parents and teachers to complete the report to establish a triangulation of data sources. Second, this study’s cross-sectional design did not allow the drawing of any cause-effective relationships among the study variables. Longitudinal studies should be conducted in the future to ascertain their deep relationships. Nevertheless, this study has shed some light on preventing school bullying and violence in rural adolescents. First, the bullies-victims group was found to have the largest number of adolescents in this study, indicating that this problem might be very serious in rural schools. The educational authorities and school administrators should pay more attention to these “double identifies” students, especially in rural schools. Second, the finding that parental behavioral control can reduce the risk of being bullies-victims implies that more proper and effective prevention programs should be developed and launched to help rural parents. This will help reduce the risk of becoming bullies-victims. Third, the quality of friends might have a more profound impact on peer victimization. Reducing affiliation with deviant peers through parental behavioral control can help prevent bullying and avoid being victimized. Fourth, more attention should be paid to girls who are bullying victims. Positive intervention measures should be taken to reduce the influence of deviant peers on girls’ victimization. Finally, home-school cooperation should be improved to reduce the number of deviant students, prevent the emergence of new deviant students, and then stop the vicious circle of bullying in time. If all these measures were put into practice, rural schools would be free of school bullying, and rural students would have better peer relationships and well-being, thus accepting educational opportunities and growing up healthily.

## 5. Conclusions

All in all, this study found that parental behavioral control was negatively correlated with deviant peer relationships, bullying, and victimization. Deviant peer affiliation was positively correlated with bullying and victimization. In terms of predictive effect, parental behavioral control could not directly predict the bullying or victimization in all groups except for the bullies/victims. The more parental behavioral control, the fewer possibilities to be bullies-victims. The mediating effect of deviant peer affiliation on victimization was moderated by gender, and deviant peer affiliation had a stronger influence on victimization in girls than that in boys.

## Figures and Tables

**Table 1:** Basic statistics for bullying and victimization (*n* = 3799).

Type	*n*	%
Bullying at Least Once	2872	76.00%
Being Bullied at Least Once	2955	77.78%
Only Bullied Others	401	10.60%
Only Being Bullied	484	12.80%
Both Bullying and Being Bullied	2471	65.40%
No-Status (Neither Bullies nor Victims)	423	11.20%

**Table 2:** Descriptive statistics and correlations for bullying, victimization, deviant peer affiliation, and parental behavioral control (*n* = 3799).

	1	2	3	4	5
1 Parental Behavioral Control	1				
2 Deviant Peer Affiliation	−0.07 **	1			
3 Bullying	−0.07 **	0.37 **	1		
4 Bullying Victimization	−0.05 **	0.35 **	0.72 **	1	
5 Gender	0.04 *	−0.29 **	−0.26 **	−0.26 **	1
M	2.83	9.61	33.01	39.25	
SD	0.90	3.43	16.77	20.21	

Notes. * *p* < 0.05, ** *p* < 0.01; In the coding of gender: male = 1; female = 2.

**Table 3:** Differences between the bullies, victims, bullies-victims, and non-status groups (*n* = 3799).

	*F*	Multiple Comparison (Significant Results)
Parental Behavioral Control	5.63 **	3 < 1; 3 < 2; 3 < 4
Number of Close Friends	7.98 ***	2 < 3 < 1; 2 < 3 < 4
Deviant Peer Affiliation	63.98 ***	3 > 1 > 4; 3 > 2

Notes. 1 = Bullies group; 2 = Victims group; 3 = Bullies-victims group; 4 = Non-status group. ** *p* < 0.01; *** *p* < 0.001.

**Table 4:** Logistic regression analyses predicting bullying and victimization grouping.

Groups	Predictors	*β*	*SE*	Wald’s *χ*^2^	*p*	Odds Ratio
Bullies	*(No-Status = Reference Group)*					
	Parental Behavioral Control	−0.02	0.07	0.06	0.81	0.98
	Deviant Peer Affiliation	0.18	0.10	3.10	0.08	1.19
	Gender	−0.26	0.15	2.95	0.09	0.77
Victims	*(No-Status = Reference Group)*					
	Parental Behavioral Control	−0.06	0.07	0.82	0.36	0.94
	Deviant Peer Affiliation	0.17	0.10	2.89	0.09	1.18
	Gender	0.10	0.15	0.48	0.49	1.11
Bullies/victims	*(No-Status = Reference Group)*					
	Parental Behavioral Control	−0.13	0.06	5.89	0.02	0.88
	Deviant Peer Affiliation	0.60	0.08	59.54	0.00	1.83
	Gender	−0.45	0.12	15.34	0.00	0.64

**Table 5:** Direct and indirect effects between parental behavioral control and bullying; victimization in the bullies-victims group (*n* = 2473).

Paths	Bullies-Victims	
	Effect	95%CI
Parental Behavioral Control → Bullying	−0.03	(−0.06, 0.01)
Parental Behavioral Control → Deviant Peer Affiliation → Bullying	−0.02	(−0.04, −0.01)
Deviant Peer Affiliation × Gender → Bullying	0.06	(−0.02, 0.14)
Parental Behavioral Control→ Victimization	−0.01	(−0.05, 0.03)
Parental Behavioral Control→ Deviant Peer Affiliation → Victimization	−0.02	(−0.03, −0.01)
Deviant Peer Affiliation × Gender→ Victimization	0.09	(0.01, 0.17)

## Data Availability

Data will be made available on request.
